# Mechanistic Insights Into Recurrent Implantation Failure: The Lactate–H3K18la–SLC7A11 Axis Explored via Endometrial Organoid and Blastoid–Endometrial Cell Implantation Models

**DOI:** 10.1111/cpr.70147

**Published:** 2025-11-16

**Authors:** Lingling Dong, Xiaobin Sun, Shiyu An, Jinfeng Xiang, Lingmin Hu, Dan Yao, Jiaqian Chang, Ruizhe Jia, Yang Yang, Shuxian Wang

**Affiliations:** ^1^ State Key Laboratory of Reproductive Medicine and Offspring Health Nanjing Medical University Nanjing Jiangsu China; ^2^ Department of Obstetrics, Women's Hospital of Nanjing Medical University Nanjing Maternity and Child Health Care Institute Nanjing Jiangsu China; ^3^ Changzhou Maternal and Child Health Care Hospital, Changzhou Medical Center Nanjing Medical University Changzhou Jiangsu China; ^4^ Department of Obstetrics and Gynecology, Zhongda Hospital, School of Medicine Southeast University Nanjing Jiangsu China; ^5^ Jiangsu Provincial Key Laboratory of Biological Therapy for Organ Failure Nanjing Medical University Nanjing Jiangsu China; ^6^ Jiangsu Environmental Health Risk Assessment Engineering Research Center, Key Laboratory of Modern Toxicology of Ministry of Education, Center for Global Health Nanjing Medical University Nanjing Jiangsu China; ^7^ Innovation Center of Suzhou Nanjing Medical University Suzhou Jiangsu China

**Keywords:** blastoid–endometrial cell implantation model, endometrial organoid, endometrial receptivity, H3K18la, lactate, recurrent implantation failure

## Abstract

Recurrent implantation failure (RIF) remains a major challenge in assisted reproductive technologies, with the underlying molecular mechanisms still largely unknown. Here, we conducted proteomic profiling and analysed publicly available single‐cell RNA sequencing data, revealing a marked decrease in lactate dehydrogenase A (LDHA) expression in RIF cases. While traditionally considered a metabolic byproduct, it is now recognised to play a role in signalling and epigenetic regulation. Utilising human endometrial organoids, we demonstrated that lactate enhances human endometrial receptivity by promoting epithelial–mesenchymal transition (EMT) and upregulating histone H3 lysine 18 lactylation (H3K18la). Further multi‐omics analyses identified solute carrier family 7 member 11 (*SLC7A11*) as an H3K18la‐regulated target. Functional assays confirmed that lactate‐induced H3K18la upregulates *SLC7A11*, thereby driving EMT and cellular migration. Notably, using a blastoid–endometrial cell implantation model, we demonstrated that SLC7A11 promotes both blastoid adhesion and expansion, highlighting its critical role in embryo–endometrial interactions. Collectively, leveraging multiple organoid systems, including endometrial organoids and blastoid–endometrial cell implantation models, our findings reveal a novel lactate–H3K18la–SLC7A11 axis that orchestrates endometrial epithelial plasticity and receptivity. In addition, this study established a robust methodological framework for investigating implantation mechanisms.

## Graphical abstract

Clinical samples first revealed impaired lactate synthesis in RIF patients. Subsequently, using endometrial organoids and a blastoid‐endometrial cell implantation model, we demonstrated that lactate enhances endometrial receptivity via H3K18la‐mediated SLC7A11 activation. These findings reveal a novel metabolism–epigenetics–function axis driving epithelial remodelling in the context of RIF.

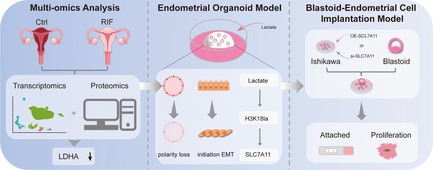

## 1 Introduction

Infertility represents a global public health challenge, affecting an estimated one in six individuals of reproductive age worldwide [1]. While assisted reproductive technologies (ART) have advanced considerably, RIF remains a major obstacle to successful pregnancy. Clinically, RIF is defined as the failure to conceive after multiple in vitro fertilisation‐embryo transfer (IVF‐ET) cycles, despite the transfer of morphologically high‐quality embryos [2, 3]. Decades of extensive basic and clinical research have yet to fully elucidate the pathophysiological mechanisms underlying RIF, underscoring an urgent need for more effective therapeutic strategies in clinical practice. The aetiology of RIF is highly multifactorial, encompassing both embryonic and endometrial factors and regulated by intricate metabolic [4], endocrine [5] and immune processes [2]. Among these, growing evidence suggests that impaired endometrial receptivity is a key contributor to RIF [6, 7], yet the molecular pathways that control this process remain unclear.

Endometrial receptivity denotes the capacity of the endometrium to permit embryo attachment and invasion within a distinct period known as the window of implantation (WOI) [8]. This transient receptive state is orchestrated by a precisely regulated hormonal environment, primarily driven by ovarian oestrogen and progesterone, which induce profound structural and molecular remodelling of the endometrium [9]. Key changes include the loss of epithelial apical‐basal polarity [10, 11], acquisition of mesenchymal‐like properties in epithelial cells [12, 13, 14] and stromal cell decidualisation [15]. These dynamic transformations are essential for successful blastocyst adhesion and implantation, thereby laying the foundation for subsequent embryonic and placental development.

Currently, oestrogen and progesterone are well‐established central regulators of endometrial receptivity. However, the metabolic reprogramming underlying the transition to a receptive state remains largely unclear. Previous studies have reported that endometrial cells exhibit enhanced glycolytic activity and increased lactate secretion during the secretory phase [16, 17], suggesting a potential link between metabolic shift and receptivity. Beyond acting as a metabolic byproduct, a recent study has revealed that lactate functions as a signalling molecule that participates in various physiological processes, including energy metabolism [18], immune modulation [19, 20], angiogenesis [21] and tumour progression [22, 23]. This multi‐functionality makes lactate an important direction for research. In mouse endometrial stromal cells, lactate has been proven to promote decidualisation. Although direct evidence connecting lactate to EMT in endometrial epithelial cells (EECs) is lacking, its capacity to induce EMT has been observed in renal epithelial cells [24, 25], raising the possibility that lactate may modulate epithelial plasticity during the WOI and thus facilitate the establishment of endometrial receptivity.

Lactate functions as a signalling molecule and exerts its biological effects through multiple pathways. Previous research has shown that lactate binds to and modulates the activity of various protein receptors, including c‐Jun, NDRG3 and PHD2 [26, 27, 28]. The expression of these receptors is linked to distinct downstream signalling cascades. In addition to its signalling role, lactate can induce lactylation, a newly identified post‐translational modification that integrates metabolic and epigenetic signalling and plays a pivotal role in the regulation of gene expression and cell fate decision [29, 30]. In endometrial cells, histone lactylation has been implicated in critical reproductive processes, including the establishment of the maternal–foetal interface [31] and stromal cell decidualisation [32]. These results suggest that lactylation may represent a key mechanism by which lactate regulates EEC function.

Our study found that lactate synthesis is reduced in patients with RIF and that lactate enhances EECs receptivity by regulating cell polarity and EMT progression. We further show that lactate induces H3K18la in EECs. Through integrating multi‐omics analyses and experimental validation, we reveal that lactate‐induced H3K18la upregulates the expression of *SLC7A11*, while deprivation of lactate under receptive conditions significantly reduces *SLC7A11* expression, indicating that *SLC7A11*, a cystine/glutamate antiporter, serves as a downstream target of H3K18la. Modulation of *SLC7A11* expression markedly affected EMT marker expression and cell migratory capacity. Importantly, through co‐culture experiments with blastoids and EECs, we functionally validated that *SLC7A11* regulates both the adhesion of blastoids to EECs and further blastoid growth. Alterations in *SLC7A11* expression significantly affected blastoid–epithelial interactions, confirming its essential role in mediating successful implantation.

Altogether, our findings uncover a novel lactate–H3K18la–SLC7A11 axis that regulates EEC plasticity and receptivity, offering new insights into epigenetic regulation and potential therapeutic targets to improve embryo implantation outcomes in cases of RIF.

## 2 Materials and Methods

### 2.1 Ethics Statement

Endometrial samples used for proteomic analysis were obtained from Changzhou Maternity and Child Health Care Hospital. This study was approved by the Ethics Committee of Changzhou Maternity and Child Health Care Hospital (approval number [2023]‐038). Endometrial tissue samples for organoid derivation were collected from Nanjing Maternity and Child Health Care Hospital. This study was approved by the Medical Ethics Committee of Nanjing Medical University (approval number [2021]‐645).

### 2.2 Baseline Characteristics of the Population

This study was collected at Changzhou Maternity and Child Health Care Hospital between 2019 and 2023. A total of 12 women undergoing assisted reproductive treatment were enrolled and stratified into two groups: the control group (*n* = 6), consisting of patients who achieved pregnancy after a single transfer of a high‐quality embryo and the RIF group (*n* = 6), defined as patients who failed to conceive after transferring ≥ 4 high‐quality embryos across at least 2 cycles. The baseline characteristics of the two groups of patients were comparable (mean age: 29.0 ± 3.06 vs. 29.83 ± 4.88 years, BMI: 21.0 ± 1.59 vs. 21.17 ± 3.47 kg/m^2^) (Table S1). Women of advanced maternal age, with obesity or with known endocrine disorders were excluded.

### 2.3 Proteomics Analysis

Proteins were extracted using SDT lysis buffer, followed by enzymatic digestion with trypsin. The peptides were gradient eluted on a C18 column and analysed on an EASY‐nLC 1200 UHPLC system coupled to a Q Exactive series mass spectrometer. High‐confidence peptide‐spectrum matching (PSM) with a confidence score > 99% was applied to ensure data quality. Proteins were identified only if at least one unique peptide was detected, and all identifications were filtered at a false discovery rate (FDR) ≤ 1.0%. Protein annotation was derived from the UniProt database. The resulting protein expression matrix underwent quality control and preprocessing prior to differential expression analysis. To handle missing values, they were first imputed using the MinDet method, and the resulting expression matrix was then log 2‐transformed to stabilise variance.

Differential expression analysis between experimental groups (RIF vs. Ctrl) was analysed using the *t*‐test. Proteins were considered differentially expressed if they satisfied both biological and statistical thresholds (fold change > 1.5 and *p* < 0.05).

### 2.4 Construction and Treatments of Endometrial Organoids (EMOs)

Donors were between 20 and 42 years of age, have a regular menstrual cycle, no histopathological abnormalities. Moreover, these donors must not have received hormone therapy for at least 3 months before sampling. Samples were preserved in PBS supplemented with 1% penicillin/streptomycin and processed for cell isolation within 3 h of collection. They were minced into small fragments using a sterile scalpel, digested in collagenase I (Solarbio) for 30 min, and resuspended in 70% Matrigel (Corning) solution and inoculated in a 48‐well plate.

According to previous reports, EMOs were cultured in 10 nM E2 to simulate the proliferative phase, and E2, 1 μM P4 (Sigma) and 1 μM cAMP (Selleck) to simulate the secretory phase. Lactate (Santa Cruz) was used at 10 mM, and GSK2837808A (Selleck) at 10 μM.

### 2.5 Detection of l‐Lactate Content

l‐Lactate levels in conditioned media were detected using a commercial lactate assay kit, per the instructions. Absorbance was measured at 530 nm. All assays were performed in biological triplicates.

### 2.6 Ishikawa Cells Culture and Treatment

Ishikawa cells (Procell) were cultured in DMEM with 10% foetal bovine serum (Hyclone) under standard conditions (5% CO_2_, 37°C). Lactate was used at 10 mM.

### 2.7 Knockdown and Overexpression of *SLC7A11*

Human *SLC7A11* siRNAs (si‐1/2/3) and control vectors were purchased from Youkang Biotech (Table S2). For overexpression, the pcDNA 3.1^+^ vector was used. Transfections were performed in 80% confluent Ishikawa cells using TransIntro EL reagent (Transgen) per manufacturer's instructions.

### 2.8 Immunofluorescent Staining

The collected samples were fixed in 4% paraformaldehyde for 30 min. They were blocked with 0.25% Triton X‐100 and 2.5% donkey serum for 1 h. Then, the primary antibody (Table S3) was incubated at 4°C overnight. Then, the secondary antibody was incubated at room temperature for 2 h, followed by nuclear staining with DAPI for 10 min and then photographed (ZEISS LSM700, Germany).

### 2.9 qRT‐PCR Analysis

After extracting RNA from the collected samples, the RNA concentration was detected by spectrophotometry. The RNA was then reverse transcribed and finally subjected to qRT‐PCR. The primer sequences were shown in Table S4. The experimental data were calculated by the 2^−ΔΔCt^ method.

### 2.10 Western Blot (WB) Analysis

Following heat denaturation, proteins were separated on SDS‐polyacrylamide gels and transferred to polyvinylidene difluoride membranes. After incubation with 5% BSA for an hour, the primary antibody (Table S3) was incubated overnight at 4°C. Then, the secondary antibody was incubated for an hour. The protein bands were photographed using an imaging system (ChemiDoc XRS, Bio‐Rad) and analysed using ImageJ software.

### 2.11 Integration Analysis of ChIP‐Seq and RNA‐Seq

H3K18la ChIP‐seq data were obtained from the GEO database with the accession number GSE207814. Raw data were first filtered with Cutadapt (v1.18) and Trimmomatic (v0.38) to remove adapter contamination, low‐quality bases, and short reads. Clean reads were then aligned to the reference genome (hg38) using Bowtie2 (v2.3.4.1), generating sorted BAM files for downstream analysis.

Using MACS2 (v2.1.2), we identified regions of significant enrichment to define peaks. The resulting broadPeak files were loaded into R (v4.1.0) and converted into genomic range objects using the GenomicRanges package. We annotated peak regions to their nearest genes based on genomic coordinates with the ChIPseeker package (v1.30.3). Finally, we visualised read alignments and peak profiles in integrative genomics viewer (IGV) (v2.9.4) to assess peak quality and genomic distribution.

For *de novo* and known motif discovery, we performed motif enrichment analysis using HOMER (v4.11) with default settings. Enriched transcription factor (TF) binding motifs were identified within the peak regions, and their positional enrichment was examined.

Bulk RNA‐seq data were obtained from the GSE192358 dataset [33]. Raw data were trimmed and quality‐filtered using Trim Galore (v0.6.7), and aligned to the reference genome using HISAT2 (v2.2.1). Subsequently, the read counts were quantified using FeatureCounts.

We used the DESeq2 package (v1.34.0) in R (v4.4.3) to identify DEGs (fold change > 2 and adj. *p* < 0.05).

### 2.12 Wound Healing Assay

Ishikawa cells were inoculated into plates. After 24 h of culture, the cells were scraped to form a straight line using a 10 μL pipette tip. After washing with PBS three times, serum‐free medium was added and the drawn straight line was photographed at different times. Wound closure was quantified using ImageJ.

### 2.13 scRNA‐Seq Analysis

scRNA‐seq data analysis results of this study were obtained from the publicly available GEO dataset GSE183837 [2]. Further quality control was applied to retain cells expressing between 500 and 6000 genes, and with mitochondrial gene content below 15%. After filtering, a total of 22,670 genes and 35,242 high‐quality cells were retained, comprising 10,737 cells from control and 24,505 cells from RIF.

Putative doublets were identified and removed using the DoubletFinder package. Each dataset was independently normalised using Seurat's NormalizeData function Cells were clustered using the FindClusters function with a resolution parameter of 0.5, resulting in four transcriptionally distinct clusters. DEGs analysis between RIF and control epithelial cell subsets was performed using the FindMarkers function with the Wilcoxon rank‐sum test, a minimum percentage expression threshold of 10%, and a log fold‐change cutoff of 0.25.

### 2.14 Construction of Human Blastoids and Blastoid–Endometrial Cell Implantation Models

Primed human UE017C1 induced pluripotent stem cells (iPSCs; generously provided by Prof. Guangjin Pan) were used to generate 8‐cell‐like cells (8CLCs). 8CLCs were detached into single cells and inoculated on AggreWell400 plates (STEMCELL Technologies). After 24 h, the medium was changed to PALLY medium [34]. After 3 days of culture, it was changed to LY medium [34].

On Day 6, blastoids were collected and co‐cultured with Ishikawa cells for 3 additional days in IVC medium. Following co‐culture, media from wells containing either attached or unattached blastoids were harvested, and the resulting supernatant was used to assess human chorionic gonadotropin (hCG) secretion using early pregnancy test strips.

### 2.15 Statistical Analysis

Data presented were expressed as mean ± SEM. Statistical graphs were generated using GraphPad Prism (version 9). Statistical analyses were conducted using Student's *t*‐tests or one‐way ANOVA, with significance thresholds of *p < 0.05, **p < 0.01, ***p < 0.001, ****p < 0.0001.

## 3 Results

### 3.1 Proteomic and Transcriptomic Profiling Reveals Suppressed Glycolysis and Impaired Lactate Production in RIF Endometrium

**FIGURE 1:**
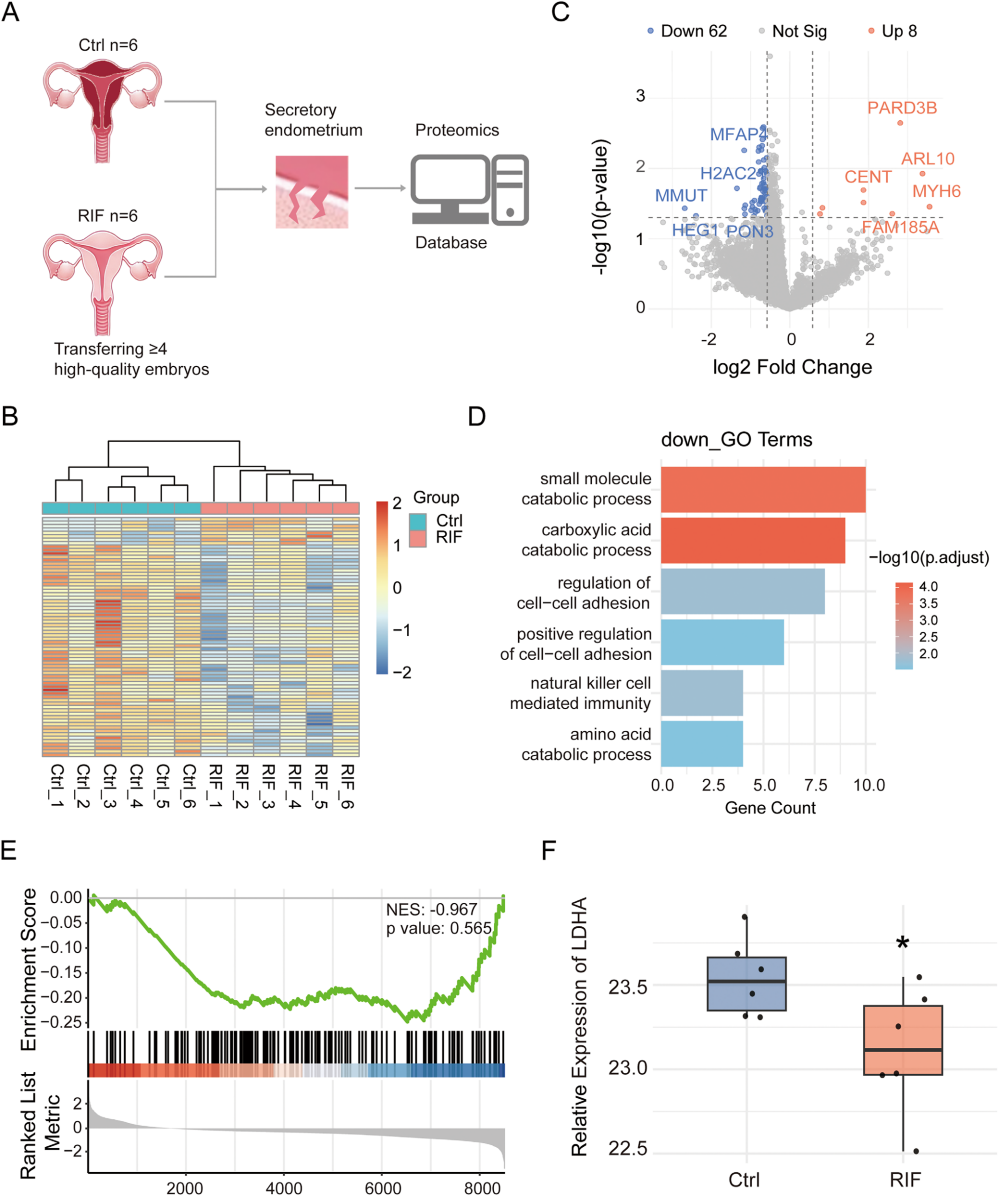
Endometrial proteomic alterations in recurrent implantation failure patients versus healthy controls. (A) Schematic overview of proteomics study design. (B) Heatmap showing differential protein expression profiles between RIF and Ctrl groups. (C) Volcano plots depicting significantly DEPs between groups. (D) Enrichment of GO terms of down‐regulated DEPs in RIFs. (E) GSEA of RIF for genes related to the glycolysis pathway. (F) Boxplot showing significantly decreased expression of LDHA in RIF endometrium. *p < 0.05.

To explore the molecular mechanisms underlying RIF, we conducted a proteomic analysis of endometrial tissue obtained from a clinically stratified cohort (*n* = 12). The cohort comprised six women diagnosed with RIF (failure to conceive after ≥ 4 high‐quality embryo transfers across 2 cycles) and six fertile controls (pregnancy achieved after a single high‐quality embryo transfer). Both groups were carefully matched for baseline characteristics (mean age: 29.0 ± 3.06 vs. 29.83 ± 4.88 years; BMI: 21.0 ± 1.59 vs. 21.17 ± 3.47 kg/m^2^), with the exclusion of advanced maternal age, obesity, or endocrine disorders to minimise confounding variables (Figure 1A).

Following preliminary matrix normalisation and missing value imputation (MVI), a total of 70 differentially expressed proteins (DEPs) were identified. Among these, eight proteins were increased and 62 were decreased in RIF. The overall proteomic alterations in RIF endometrium were visualised using hierarchical clustering heatmaps and volcano plots (Figure 1B,C). Previous studies have reported impaired adhesion capacity in RIF, accompanied by significant downregulation of the adhesion‐related factor MFAP4 [35]. Consistently, our cohort data demonstrated the same pattern of reduced MFAP4 expression (Figure 1C).

Gene ontology (GO) analysis revealed that biological processes closely associated with endometrial receptivity—such as regulation of cell–cell adhesion and natural killer cell‐mediated immunity—were significantly suppressed in the RIF group (Figure 1D). In addition, metabolic processes, including the carboxylic acid catabolic process, particularly linked to carbohydrate metabolism, were also markedly downregulated. As active glycolysis is recognised as a hallmark of endometrial receptivity during implantation, our gene set enrichment analysis demonstrated the global downregulation of glycolysis‐related pathways in RIF patients (Figure 1E). Furthermore, we observed a robust reduction in the expression of LDHA, both in our proteomic data (Figure 1F).

To further validate our findings, we performed an integrated analysis of publicly available scRNA‐seq datasets, comprising 60, 222 cells from three fertile controls and six patients with RIF [2]. All sequenced cells were categorised into four major cell populations based on canonical marker genes: fibroblast‐like cells (FIB; expressing *COL1A1* and *VIM*), epithelial cells (EC; expressing *EPCAM*, *KRT8* and *KRT18*), immune cells (IC; expressing *PTPRC* and *CD3D*) and vascular cells (VASC; expressing *CLDN5* and *VWF*) (Figure S1A,B). Consistent with our proteomic findings, the expression of *LDHA* was markedly reduced in the endometrium of RIF patients at the single‐cell level (Figure S1C,D). Notably, stratified analysis showed that *LDHA* expression was robustly reduced in EC, FIB and IC from RIF patients compared to controls, with the most pronounced difference observed in ECs (Figure S1E).

Collectively, these proteomic and transcriptomic analyses suggest that reduced lactate synthesis is a defining feature of the RIF endometrium and may contribute to infertility in this population.

### 3.2 Lactate Drives Establishment of Endometrial Receptivity in EMOs

**FIGURE 2:**
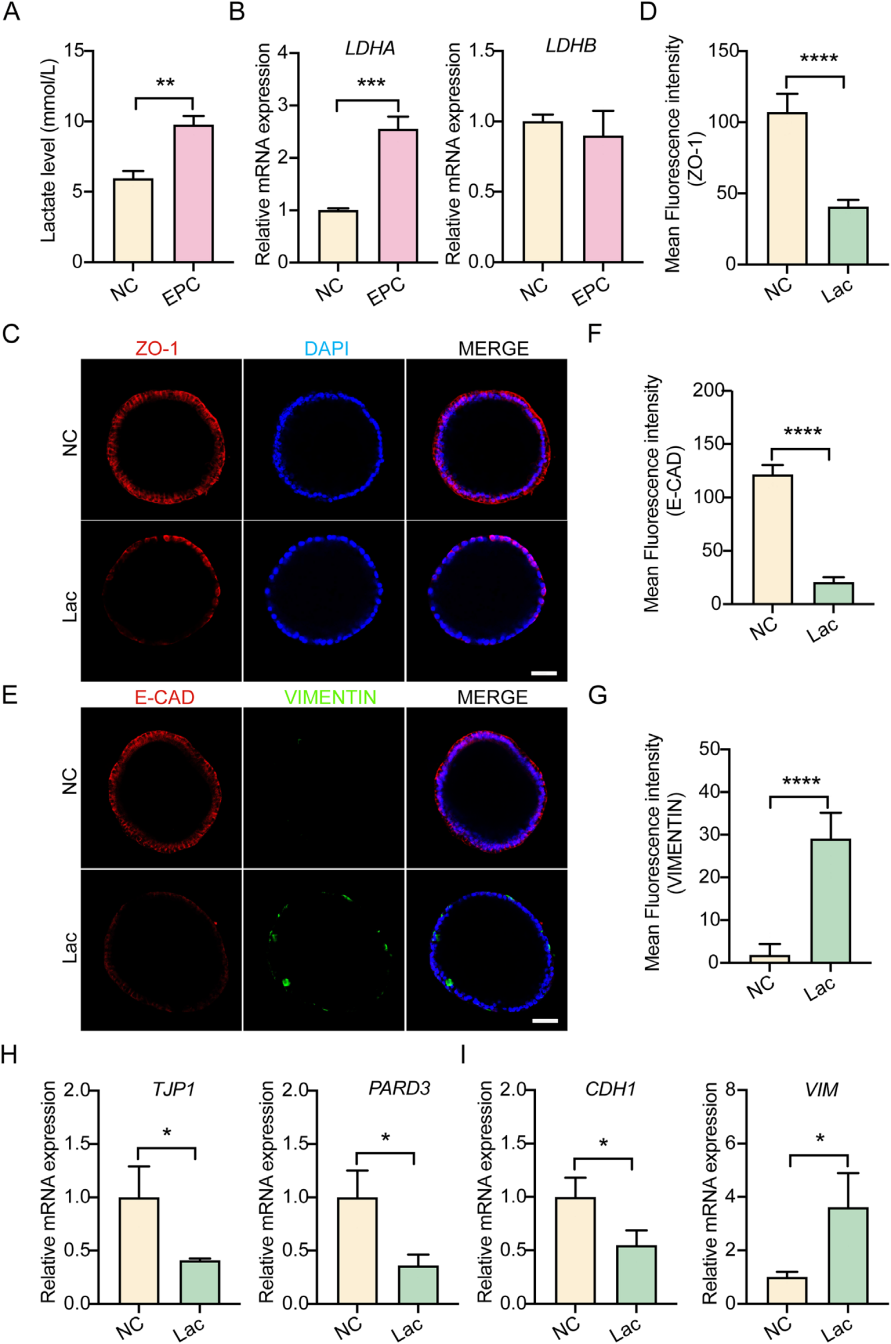
Lactate disrupts epithelial polarity and induces EMT in human EMOs. (A) Quantification of lactate concentration in the culture supernatant of EMOs following combined EPC treatment. (B) Analysis of *LDHA* and *LDHB* mRNA expression levels in EMOs following EPC treatment. (C and D) Representative immunofluorescence staining and mean fluorescence intensities statistics of ZO‐1 in human EMOs treated or untreated with lactate (Lac). Scale bar, 50 μm. *n* = 12. (E–G) Immunofluorescence images and mean fluorescence intensities statistics of EMT markers E‐CAD and VIMENTIN in NC and Lac‐treated EMOs. Scale bar, 50 μm. *n* = 12. (H and I) Analysis of polarity‐associated genes *TJP1* and *PARD3*, EMT marker genes *VIM* and *CDH1*, in Lac‐treated and untreated EMOs. *p < 0.05, **p < 0.01, ***p < 0.001, ****p < 0.0001.

Building on our findings that diminished glycolytic activity and lactate synthesis are prominent features of the RIF endometrium, we sought to clarify how lactate influences endometrial receptivity at the molecular and cellular levels of the endometrium. To this end, we turned to EMOs, an advanced three‐dimensional (3D) in vitro model that faithfully reproduces the structural, functional and hormonal dynamics of native human endometrial tissue. Primary endometrial tissues were isolated from healthy donors and used to generate hormone‐responsive EMOs, following the protocol previously described by Turco et al. [36] (Figure S2A). Treatment with estradiol (E2) to simulate the proliferative phase led to a significant increase in PR‐ and ER‐positive cell populations. In contrast, administration of E2 combined with progesterone and cAMP (EPC), which mimics the secretory phase, resulted in a marked reduction in PR‐ and ER‐positive cells compared to E2 treatment, indicating that EMOs robustly recapitulate hormonal responses of the endometrium in vivo (Figure S2B,C). These findings confirm that our organoid model reliably simulates the menstrual cycle of the endometrium, thus providing a powerful platform for studying endometrial receptivity. Based on this model, we found that secretory‐phase EMOs subjected to EPC exhibited elevated lactate levels (Figure 2A) and increased expression of *LDHA* mRNA (Figure 2B).

To further expound the role of lactate in endometrial receptivity, we cultured EMOs with lactate and assessed key markers. Immunofluorescent analysis revealed that lactate markedly decreased the expression of ZO‐1 (Figure 2C,D), closely recapitulating the phenotype observed in secretory‐phase endometrial samples. Concurrently, lactate treatment led to a significant decrease in E‐cadherin (E‐CAD) and an increase in VIMENTIN (VIM) expression (Figure 2E–G), indicative of enhanced EMT progression. These effects were corroborated by qRT‐PCR analysis, which showed a substantial downregulation of several key polarity genes, such as *TJP1* and *PARD3* (Figure 2H), while also decreasing EMT‐associated genes *CDH1* and upregulating *VIM* mRNA expression, respectively (Figure 2I). Together, these results show that lactate promotes the loss of epithelial polarity and induction of EMT in EMOs.

To establish a causal relationship, we blocked lactate production by treating EMOs with GSK 2837808A (GSK), an LDHA inhibitor, during EPC‐induced secretory‐phase modelling. Immunofluorescence analysis showed that EPC treatment markedly reduced the expression of ZO‐1 compared to controls (Figure S3A,B), consistent with the polarity loss characteristic of the secretory phase in vivo. Correspondingly, E‐CAD expression was significantly downregulated, while VIM expression was upregulated, supporting the occurrence of EMT during the acquisition of endometrial receptivity. Remarkably, inhibition of lactate production by GSK treatment substantially restored epithelial polarity and attenuated EMT progression (Figure S3A–E). Moreover, qRT‐PCR analysis revealed that the EPC‐induced alterations in polarity and EMT‐associated gene expression, such as *TJP1*, *PARD3*, *CDH1* and *VIM*, were significantly reversed by GSK co‐treatment (Figure S3F,G).

Collectively, our results suggest that lactate production is crucial for the loss of epithelial polarity and the initiation of EMT during endometrial receptivity.

### 3.3 Lactate Drives H3K18 Histone Lactylation in EMOs and Ishikawa Cells

**FIGURE 3:**
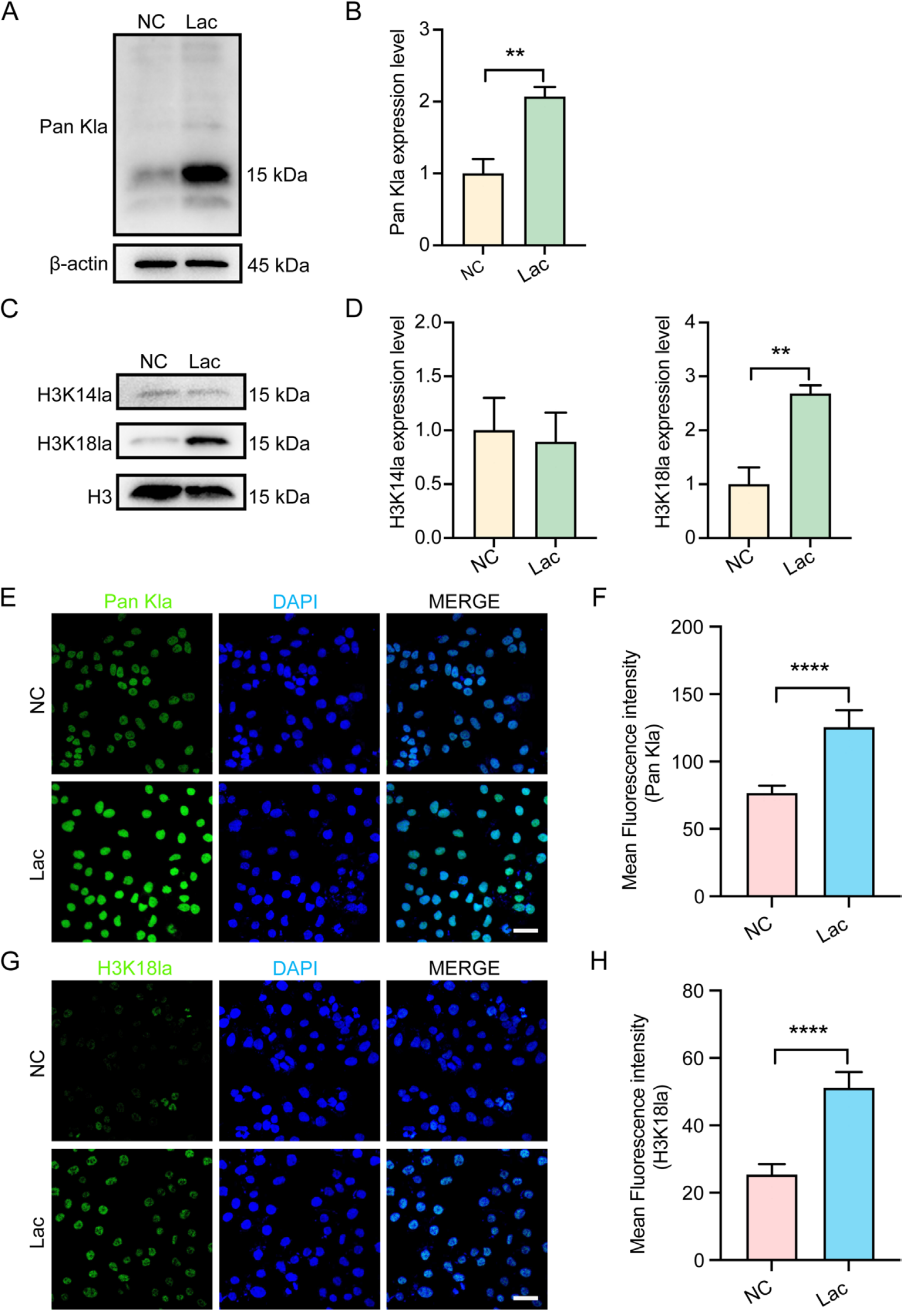
Lactate‐mediated upregulation of H3K18 lactylation in EMOs and Ishikawa cells. (A and B) WB analysis of global lysine lactylation (Pan Kla) in EMOs treated with Lac. *n* = 3. (C and D) WB analysis of histone lactylation at H3K14la and H3K18la sites in response to Lac. *n* = 3. (E and F) Representative immunofluorescence images and mean fluorescence intensities of Pan Kla. Scale bar, 50 μm. *n* = 12. (G and H) Representative immunofluorescence images and mean fluorescence intensities of H3K18la and DAPI in treated with Lac. Scale bar, 50 μm. *n* = 12. **p < 0.01, ****p < 0.0001.

Previous research has indicated that lactate drives protein lactylation [29], a post‐translational modification involved in immune regulation [19], cell differentiation [37] and embryonic development [38]. To further investigate the mechanistic role of lactate in endometrial receptivity, we performed WB analysis of lactate‐treated EMOs. Our results revealed that lactate treatment significantly increased overall lactylation (Pan kla) levels compared to control EMOs, with a prominent band observed near 15 kDa, corresponding to histone H3 (Figure 3A,B). Notably, previous reports have identified lactylation at lysine residues K14 and K18 on histone H3 in endometrial cells. Therefore, we evaluated the lactylation status of these residues (H3K18la and H3K14la) by WB. The findings demonstrated that H3K18la levels increased significantly after lactate treatment, while H3K14la levels remained largely unchanged (Figure 3C,D).

Consistent results were obtained in Ishikawa cells, which are usually utilised as a model system for investigating endometrial function and related regulatory mechanisms. Subsequently, Pan kla and H3K18la also increased significantly in lactate‐treated cells (Figure 3E–H).

These findings collectively indicate that lactate enhances H3K18la modification in both EMOs and EECs, suggesting a potential epigenetic mechanism through which lactate may modulate endometrial function.

### 3.4 Screening and Identification of H3K18la Downstream Targets

**FIGURE 4:**
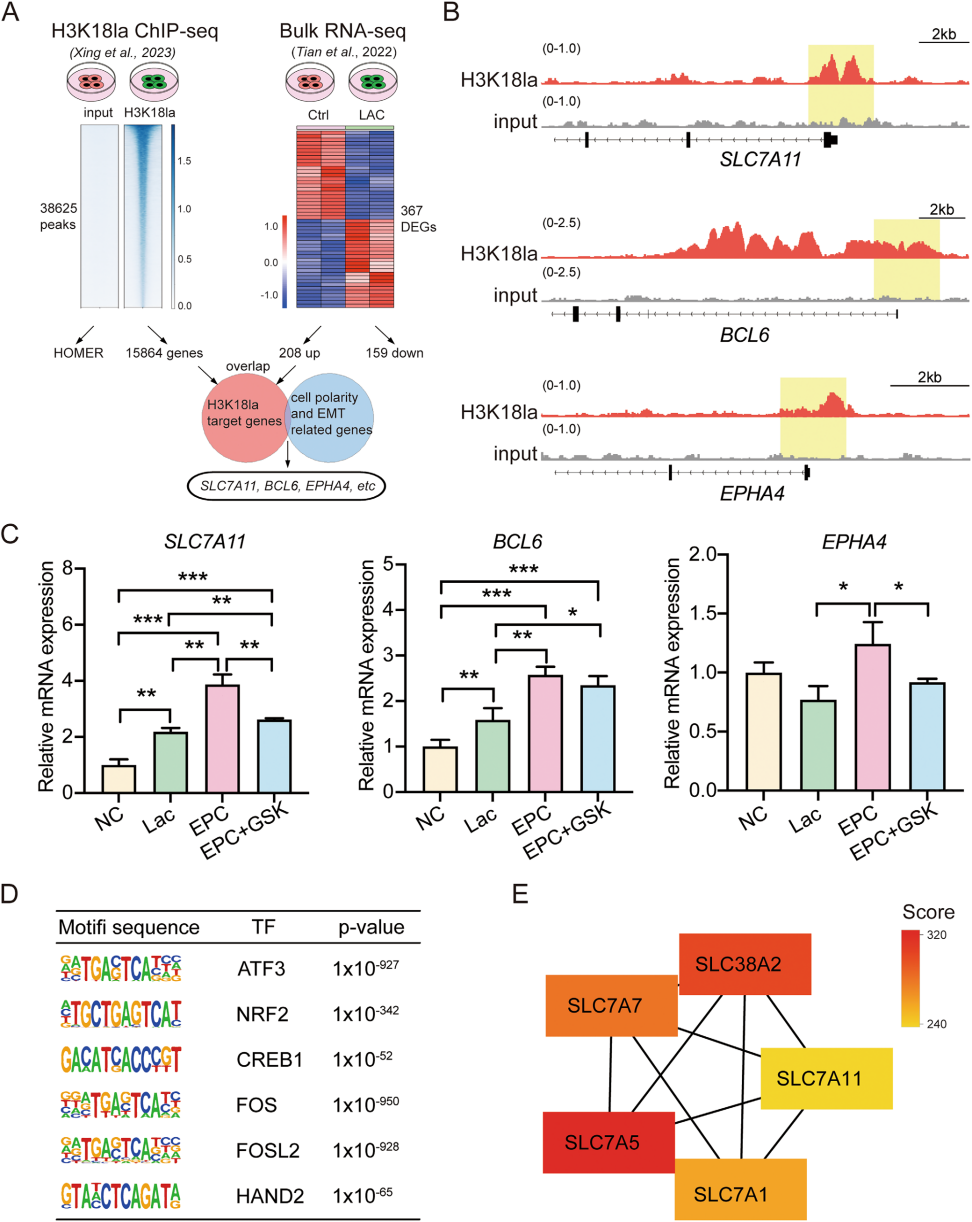
*SLC7A11* was upregulated by lactate‐mediated H3K18la modification. (A) Schematic workflow integrating H3K18la ChIP‐seq and bulk RNA‐seq. (B) ChIP‐seq signal tracks showing H3K18la enrichment at promoter or regulatory regions of *SLC7A11*, *BCL6* and *EPHA4*. Yellow boxes highlight H3K18la peak regions. (C) Analysis of *SLC7A11*, *BCL6* and *EPHA4* mRNA levels under various treatment conditions in EMOs. *p < 0.05, **p < 0.01, ***p < 0.001. (D) Motif enrichment analysis of H3K18la‐bound regions reveals several TFs potentially mediating lactate‐induced gene expression changes. (E) PPI network analysis of top‐ranked H3K18la‐regulated genes using STRING database.

Histone modification plays key roles in orchestrating transcriptional activation and repression of target genes. To elucidate the downstream regulatory mechanisms mediated by the lactate/H3K18la axis, we performed integrated analysis of publicly available ChIP‐seq and RNA‐seq datasets to identify H3K18la target genes involved in EMT. Analysis of ChIP‐seq data yielded H3K18la‐enriched 38, 625 peaks mapping to 15, 864 potential target genes, while comparative analysis with bulk RNA‐seq data from lactate‐treated samples revealed 208 significantly upregulated genes. Intersection of these datasets identified 111 consensus genes that were both H3K18la‐bound and lactate‐responsive, hereafter designated as H3K18la target genes (Figure 4A). Bioinformatic annotation revealed that this group includes several genes associated with endometrial function, notably established regulators of cell polarity and EMT, such as *SLC7A11* [39], *BCL6* [40] and *EPHA4* [41]. Notably, H3K18la exhibited pronounced enrichment at the promoter regions of *SLC7A11*, *BCL6* and *EPHA4*, strongly suggesting their direct transcriptional regulation through histone lactylation (Figure 4B).

To functionally validate these findings, we investigated the effects of lactate modulation under two experimental conditions: lactate supplementation and pharmacological lactate deprivation in EMOs. qRT‐PCR analysis demonstrated that all three candidate genes were markedly upregulated following EPC hormone treatment. Strikingly, lactate treatment selectively enhanced the expression of *SLC7A11* and *BCL6*, whereas *EPHA4* levels remained unchanged (Figure 4C). Conversely, lactate deprivation under hormone‐treated conditions led to a pronounced reduction in *SLC7A11* expression but did not significantly alter *BCL6* expression, suggesting that lactate specifically governs *SLC7A11* transcriptional regulation (Figure 4C).

Motif analysis of H3K18la ChIP‐seq data identified multiple TFs comprising the H3K18la regulatory network, including several well‐established TFs associated with uterine content receptivity, such as FOS, CREB1, FOSL2 and Hand2 (Figure 4D). Intriguingly, there were also known upstream regulators of SLC7A11 (ATF2 and NFR2). Additionally, PPI network analysis of the 111 lactate‐responsive genes pinpointed SLC7A11 as a central hub (Figure 4E), reinforcing its pivotal role in lactate‐mediated signalling.

Together, these findings establish *SLC7A11* as a principal downstream effector of the lactate/H3K18la axis, critically modulating endometrial receptivity.

### 3.5 *SLC7A11* Functions as a Key Regulator of Lactate‐Driven EMT and Enhanced Motility in Endometrial Cells

To probe the role of *SLC7A11* in the endometrial cells, we employed both loss‐ and gain‐of‐function approaches by transfecting Ishikawa cells with either *SLC7A11*‐specific siRNA or *SLC7A11* overexpression plasmids, respectively. qRT‐PCR analysis confirmed that *SLC7A11* knockdown significantly reduced its mRNA levels (Figure S4A), and WB analysis further demonstrated a marked decrease in SLC7A11 protein expression (Figure S4B,C). Conversely, overexpression of SLC7A11 resulted in significant upregulation of mRNA and protein levels, which was confirmed by qRT‐PCR and WB (Figure S4D–F). These results confirmed the successful establishment of our experimental model, enabling subsequent functional investigations.

**FIGURE 5:**
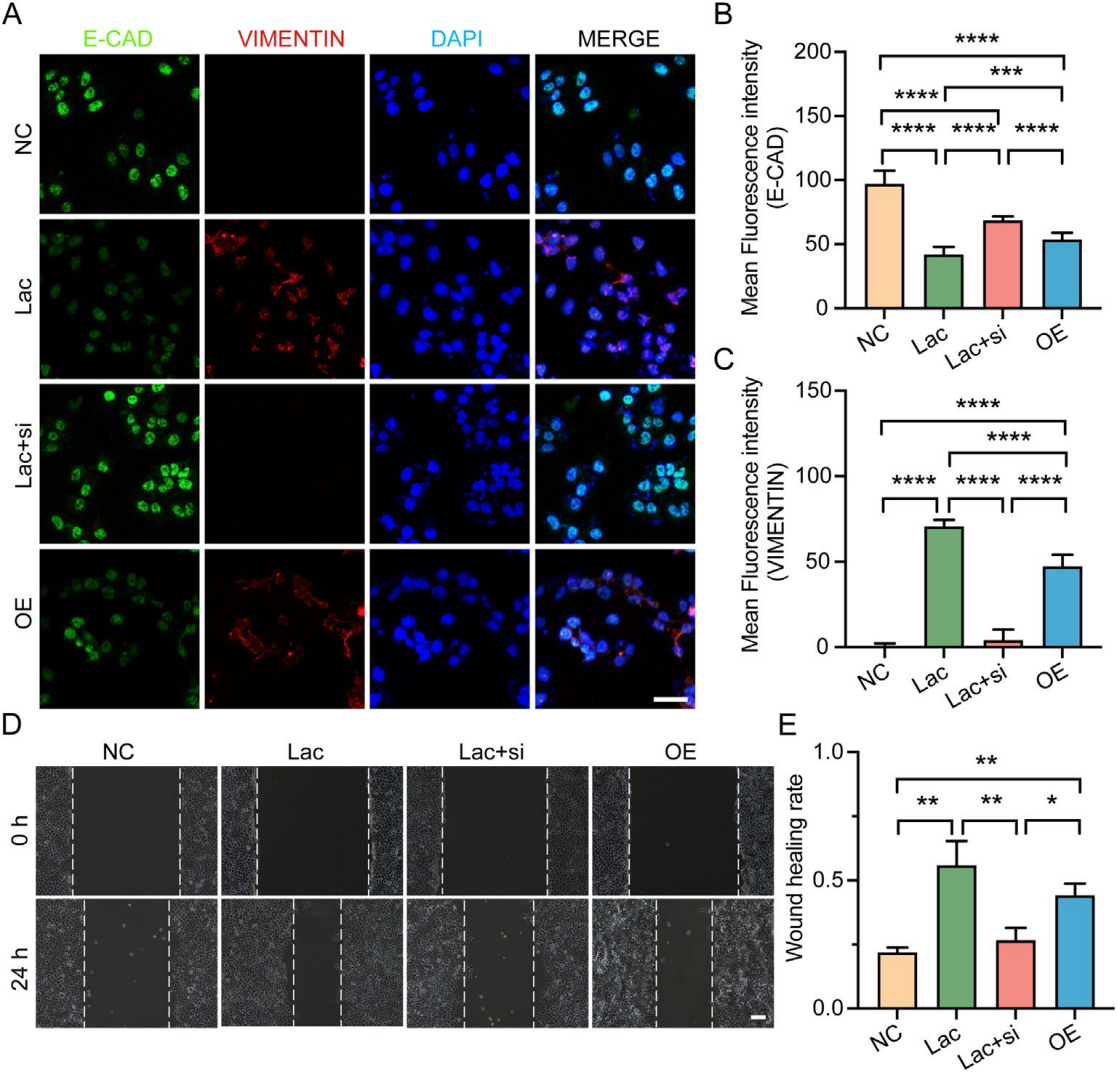
*SLC7A11* is essential for lactate‐promoted EMT and enhanced motility in Ishikawa cells. (A–C) Representative immunofluorescence images and mean fluorescence intensities of E‐CAD, VIMENTIN and DAPI in Ishikawa cells following treatment with NC, Lac, lactate plus SLC7A11 knockdown (Lac + si), or SLC7A11 overexpression (OE). Scale bars, 50 μm. *n* = 9. (D) Representative images of wound healing assay performed at 24 h after scratch formation. Scale bars, 100 μm. (E) Quantification of wound healing rates based on gap closure after 24 h. *n* = 3. *p < 0.05, **p < 0.01, ***p < 0.001, ****p < 0.0001.

Notably, immunofluorescent staining assays showed that in Ishikawa cells treated with lactate, the expression of E‐CAD was markedly reduced, while VIM expression was markedly increased, paralleling the observations in EMOs. Furthermore, compared to lactate treatment, co‐treatment with lactate and *SLC7A11* siRNA significantly restored E‐cadherin expression and markedly reduced Vimentin levels, suggesting a critical regulatory role for *SLC7A11* in lactate‐induced EMT. Likewise, overexpression of *SLC7A11* alone mirrored the effects of lactate treatment, with a pronounced reduction in E‐CAD, while VIM levels were increased (Figure 5A–C). Moreover, the wound‐healing assays, a widely used method to evaluate cellular migratory capacity [42], revealed that both lactate treatment and *SLC7A11* overexpression significantly enhanced the migratory capacity of Ishikawa cells, whereas co‐treatment with lactate and *SLC7A11* siRNA markedly impaired cell migration (Figure 5D,E).

Together, these results demonstrate that *SLC7A11* acts as a downstream target gene of lactate, playing a vital role in driving the EMT process in endometrial cells.

### 3.6 Lactate‐Driven Upregulation of *SLC7A11* Facilitates Blastoid Implantation via Enhanced Endometrial Receptivity

**FIGURE 6:**
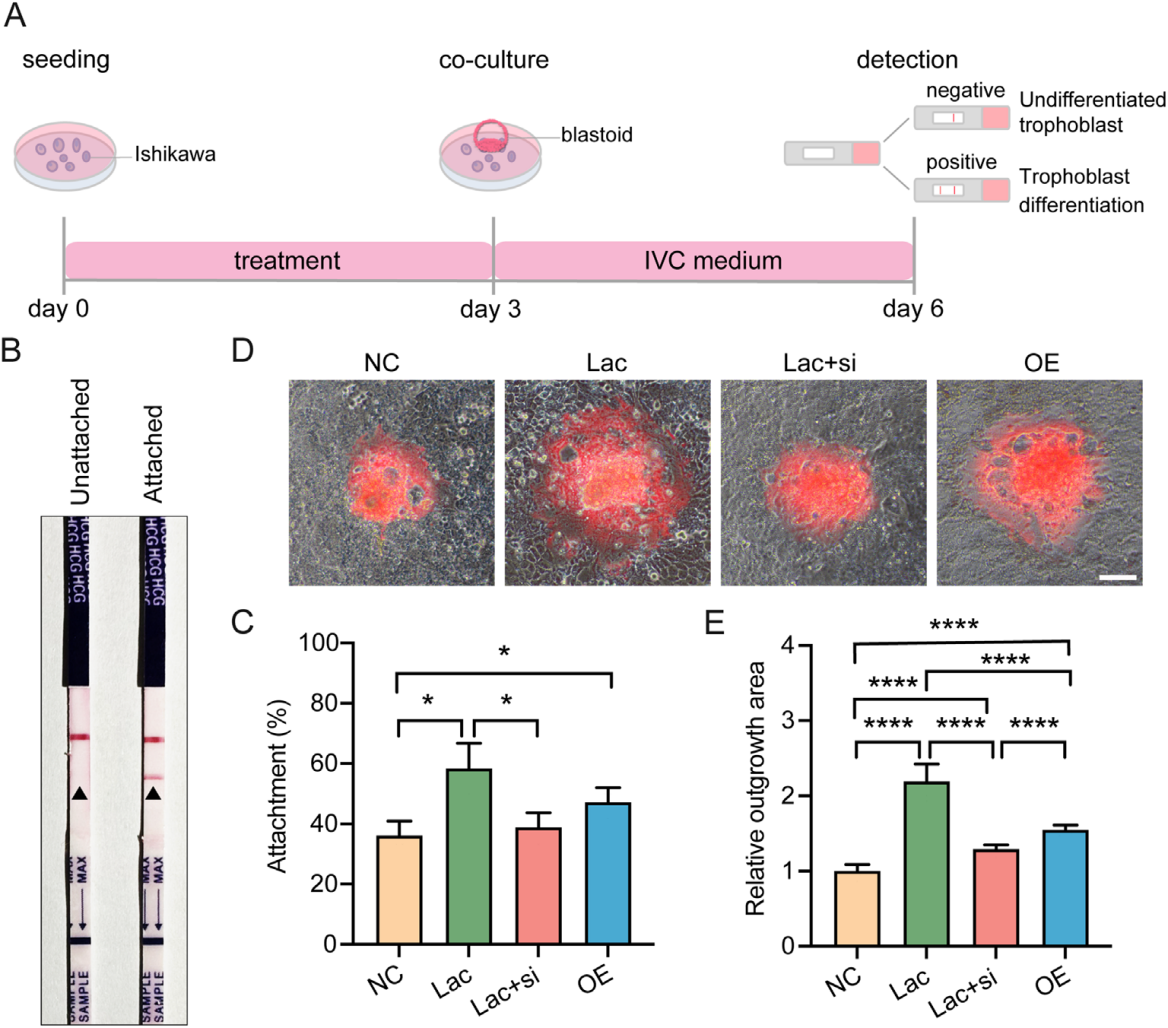
*SLC7A11* mediates lactate‐induced improvements in embryo implantation efficiency. (A) Schematic illustration of the experimental process. (B) Representative images of hCG detection strips in the culture medium for indicating blastoid attachment status in cultured 72 h. (C) Attachment efficiency of human blastoids across different treatment groups after co‐culture for 24 h. *n* = 3. (D) Representative phase‐contrast images of blastoids (tdTomato^+^) showing outgrowth morphology after 72 h co‐culture under different treatments. Scale bars, 100 μm. (E) Quantification of the relative outgrowth area of attached blastoids. *n* = 6. *p < 0.05, ****p < 0.0001.

To probe the role of *SLC7A11* in regulating endometrial receptivity, we used a previously established human blastoid model [34] and co‐cultured these blastoids with Ishikawa cells to mimic embryo implantation (Figure 6A). Briefly, tdTomato‐labelled iPS cells were induced to form 8CLCs (Figure S5A–C) and subsequently cultured in AggreWell for 6 days to generate blastoid structures resembling human blastocysts in both morphology and lineage specification (Figure S5D–F). These blastoids were subsequently co‐cultured with Ishikawa cells in a system we designated as the blastoid–endometrial cell implantation model (BEIM). This model allowed us to evaluate both the efficiency of blastoid attachment and the extent of blastoid proliferation. After 72 h of co‐culture, only the attached blastoids secreted hCG, whereas unattached blastoids did not (Figure 6B), indicating that blastoids capable of adhering to Ishikawa cells had initiated implantation‐like proliferation and differentiation, analogous to events in utero. This model system offers a physiologically relevant platform for studying embryo–endometrium interactions.

Using the BEIM, we further assessed the effects of different treatments on blastoid attachment efficiency. After 24 h, both lactate treatment and *SLC7A11* overexpression in Ishikawa cells significantly increased the proportion of attached blastoids compared to controls (Figure 6C). This enhancement was notably reversed upon co‐treatment with lactate and *SLC7A11* siRNA. To further explore how *SLC7A11* influences blastoid proliferation during implantation, we measured the area of tdTomato‐labelled blastoids at 72 h of co‐culture. The results showed that both lactate treatment and *SLC7A11* overexpression led to a significant enlargement of blastoid area, whereas this effect was attenuated by *SLC7A11* knockdown via siRNA (Figure 6D,E). These results showed that SLC7A11 plays an important role in promoting blastoid attachment and proliferation.

Overall, our results suggest that lactate enhances H3K18la modification in endometrial cells, which subsequently upregulates *SLC7A11* expression to improve endometrial receptivity and facilitate embryo implantation.

## 4 Discussion

Through integrative proteomic and scRNA‐seq analyses of endometrial samples from RIF, we identified aberrant lactate synthesis in endometrium as a distinct molecular feature of the RIF endometrium. Although the number of RIF samples included in the proteomic analysis was limited due to strict inclusion criteria, this study nevertheless uncovered lactate as a pivotal regulator in establishing human endometrial receptivity. Previous research has reported that lactate promotes decidualisation of mouse endometrial stromal cells [32], however, its specific effects on human EECs have remained largely unexplored. In this study, using a hormone‐responsive human EMOs model, we demonstrated that lactate significantly enhances EEC receptivity by driving EMT and upregulating H3K18la levels. Mechanistically, we identified *SLC7A11* as a pivotal downstream target of H3K18la, mediating the lactate‐induced EMT process and facilitating embryo–endometrial interactions. Our findings reveal a previously unrecognised epigenetic mechanism whereby lactate, through histone lactylation at H3K18, modulates endometrial receptivity. The elucidated lactate–H3K18la–*SLC7A11* axis constitutes a novel regulatory pathway and represents promising therapeutic targets for improving implantation success in ART.

While IVF–EVT has emerged as a cornerstone of infertility treatment, approximately 10% of patients still suffer from RIF, a major clinical challenge in ART with unclear pathophysiology [43]. Our study identifies impaired lactate biosynthesis as a molecular hallmark of endometrial dysfunction in RIF, positioning lactate both as a mechanistic contributor to receptivity and as a tractable clinical readout. Current embryo transfer protocols rely on endometrial thickness (≥ 7 mm) and hormonal thresholds to time transfer, yet these measures often miss subtle but clinically meaningful shifts in the endometrial microenvironment. Recognising lactate's protective and homeostatic roles in endometrial tissue, our data support lactate as a candidate signature of impaired receptivity in RIF. Given that lactate can be quantified rapidly and with minimal invasiveness, we propose its evaluation as a pre‐transfer biomarker. If validated in prospective studies, integrating lactate‐based metrics with existing protocols could improve IVF‐ET success rates, reduce unnecessary cycles and enable more precise, biology‐informed reproductive care.

Historically regarded as a glycolytic by‐product, lactate is now understood to function as a signalling molecule across diverse biological contexts [44, 45]. In line with this paradigm, our integrated proteomic and transcriptomic analyses revealed impaired lactate synthesis in the endometrium of RIF patients (Figures 1 and S1), implicating lactate as a potential signalling mediator in the pathogenesis of implantation failure. In our EMOs model, lactate production and LDHA expression significantly increased during the secretory phase (Figure 2A,B), consistent with previous findings on metabolic reprogramming during the implantation window [16, 17]. Pharmacological deprivation of lactate restored epithelial polarity and suppressed EMT features (Figure S3A–G), highlighting the critical role of lactate‐driven remodelling during implantation. During this process, receptive EECs undergo partial depolarisation and transient EMT, which facilitate embryo penetration through the epithelial barrier and are considered prerequisites for successful attachment [14, 46]. Notably, this loss of polarity represents a finely regulated and reversible depolarisation, rather than a complete breakdown of epithelial integrity or a terminal cellular transition. Unlike the irreversible EMT observed in metastatic cancers, these structural changes are transient and reversible. Such dynamic remodelling of epithelial polarity may represent an essential physiological mechanism enabling the endometrium to become transiently receptive to embryo implantation [10, 12, 13].

At the epigenetic level, our study reveals that lactate selectively induces histone lactylation at the H3K18 site in endometrial cells. This type of epigenetic modification has recently been suggested to link cellular metabolic states to gene regulatory programmes, particularly in response to lactate accumulation. Our data demonstrate that H3K18la levels are significantly elevated following lactate treatment, while H3K14la remains unchanged (Figure 3C). This site‐specific modification was observed not only in EMOs but also in Ishikawa cells (Figure 3G,H), suggesting a unique regulatory role for H3K18la in the epigenetic landscape of the endometrium. These findings were consistent with previous reports highlighting the crucial role of site‐specific lactylation patterns in reproductive tissues [31, 47, 48].

Besides, we also identify *SLC7A11*, a cystine/glutamate antiporter involved in redox homeostasis and cellular metabolic balance [49, 50], as a critical downstream target of H3K18la in EECs. Beyond its well‐established role as an antioxidant regulator and gatekeeper of ferroptosis [51], *SLC7A11* has been implicated in EMT and cell migration in tumour cells [39, 52]. In our study, lactate‐induced H3K18la enrichment in the *SLC7A11* promoter region resulted in its transcriptional upregulation, which in turn promoted EMT in EMOs. Functional studies with *SLC7A11* overexpression and knockdown confirmed its necessity for lactate‐mediated EMT induction, as evidenced by changes in classic EMT markers and cell migration behaviour (Figure 5). These results indicate that *SLC7A11* functions as a pivotal effector linking lactate metabolism and histone modification to epithelial plasticity during the acquisition of endometrial receptivity. Notably, *SLC7A11* operates not only as a key plasma membrane transporter but also as a non‐classical proton transporter located on the lysosomal membrane, conferring dual roles in antioxidant defence and regulation of lysosomal activity [53, 54]. Since both processes are crucial for embryo implantation [55, 56], *SLC7A11* likely exerts multifaceted regulatory effects on endometrial receptivity and embryo implantation through these mechanisms. Supporting this link, activation of the LIF‐STAT3 pathway has been reported to enhance lysosomal function in luminal epithelial cells, providing additional mechanistic evidence for *SLC7A11's* role in lysosomal regulation [56, 57].

Consistent with a role in embryo–endometrium crosstalk, co‐culture of blastoids with EECs showed that modulating *SLC7A11* affected blastoid attachment and expansion (Figure 6). These findings highlight *SLC7A11* as the central mediator of the lactate‐driven epigenetic and metabolic reprogramming required for successful implantation. However, while the BEIM successfully recapitulates key implantation events such as adhesion and growth, several limitations remain. For instance, the endometrial epithelial component in BEIM consists of Ishikawa cells, a carcinoma‐derived line, that may not fully represent the physiological state of native EECs or the typical columnar architecture of the endometrial epithelium, and the absence of stromal cells in this model restricts the representation of maternal–embryonic crosstalk. Future work should refine these models and integrate insights with physiologically relevant animal systems, such as primates [58] and guinea pigs [59], to achieve a more comprehensive understanding of the complex process of human embryo implantation. With continuous innovation in model systems and research technologies [60, 61], such efforts will strengthen the translational path towards biomarker‐guided, mechanism‐based interventions, including the clinical evaluation of lactate as a pre‐transfer indicator of endometrial receptivity.

Together, our findings uncover a previously unrecognised regulatory network in which lactate‐driven histone lactylation modulates endometrial receptivity, with *SLC7A11* acting as a central effector. This mechanistic link between lactate metabolism, histone lactylation and endometrial receptivity not only deepens understanding of embryo implantation biology but also highlights novel therapeutic targets for improving reproductive outcomes in patients with RIF or endometrial dysfunction. Further studies are necessary to investigate the broader epigenomic landscape of lactylation in its potential interplay with hormonal signalling pathways in the human endometrium.

## Author Contributions

Conceptualisation: Yang Yang and Lingling Dong. Methodology: Lingling Dong and Xiaobin Sun. Investigation: Lingling Dong, Xiaobin Sun, Shiyu An, Jinfeng Xiang, Lingmin Hu, Dan Yao and Jiaqian Chang. Data curation: Lingling Dong and Xiaobin Sun. Visualisation: Lingling Dong. Supervision and funding acquisition: Yang Yang, Ruizhe Jia, Shuxian Wang and Shiyu An. Writing – original draft: Lingling Dong and Shiyu An. Writing – review and editing: Yang Yang, Lingling Dong, Shuxian Wang and Shiyu An.

## Conflicts of Interest

The authors declare no conflicts of interest.

## Supporting information


**Data S1:** Supporting Information.

## Data Availability

The data that support the findings of this study are available on request from the corresponding author. The data are not publicly available due to privacy or ethical restrictions.
