# Supplementary material for: Mechanistic Insights Into Recurrent Implantation Failure: The Lactate–H3K18la–SLC7A11 Axis Explored via Endometrial Organoid and Blastoid–Endometrial Cell Implantation Models
Source: Cell Prolif. 2025 Nov 16;59(6):e70147. doi: 10.1111/cpr.70147 (PMC13241822; doi:10.1111/cpr.70147)
Supplement: Supplementary file 1 — Data S1: Supporting Information. [file CPR-59-e70147-s001.docx]

Supplementary Information

**Mechanistic Insights into Recurrent Implantation Failure: The Lactate-H3K18la-SLC7A11 Axis Explored via Endometrial Organoid and** **Blastoid-Endometrial Cell Implantation Models**

Lingling Dong^1†^, Xiaobin Sun ^1†^, Shiyu An^1^, Jinfeng Xiang^2^, Lingmin Hu^3^, Dan Yao^2^, Jiaqian Chang^3^, Ruizhe Jia^4*^, Yang Yang^1,5,6,7*^, Shuxian Wang^3*^

**Author address:**

^1^ State Key Laboratory of Reproductive Medicine and Offspring Health, Nanjing Medical University, Nanjing, 211166, Jiangsu, China.

^2^ Department of Obstetrics, Women’s Hospital of Nanjing Medical University, Nanjing Maternity and Child Health Care Institute, Nanjing, 210004, Jiangsu, China.

^3^ Changzhou Maternal and Child Health Care Hospital, Changzhou Medical Center, Nanjing Medical University, Changzhou, 213016, Jiangsu, China.

^4^ Department of Obstetrics and Gynecology, Zhongda Hospital, School of Medicine, Southeast University, Nanjing 210000, Jiangsu, China.

^5^ Jiangsu Provincial Key Laboratory of Biological Therapy for Organ Failure, Nanjing Medical University; Nanjing 211166, Jiangsu, China.

^6^ Jiangsu Environmental Health Risk Assessment Engineering Research Center, Key Laboratory of Modern Toxicology of Ministry of Education, Center for Global Health, Nanjing Medical University; Nanjing 211166, Jiangsu, China.

^7^ Innovation Center of Suzhou Nanjing Medical University, Suzhou, 215000, Jiangsu, China.

^†^ These authors contributed equally to this work.

*Corresponding authors.

**Emails of authors:**

Lingling Dong: llingdong24@163.com

Xiaobin Sun: xbinsun@126.com

Shiyu An: 839730691@qq.com

Jinfeng Xiang: xjf19990221@163.com

Lingmin Hu: hulingmin1986@126.com

Dan Yao: 1134852276@qq.com

Jiaqian Chang: JeaquinaChang@outlook.com

Ruizhe Jia: rzjia9599@163.com

Yang Yang: yangyang11@njmu.edu.cn

Shuxian Wang: wsxnju95@163.com

**This PDF file includes:**

**Figures. S1 to S5**

**Tables. 1 to 4**

**
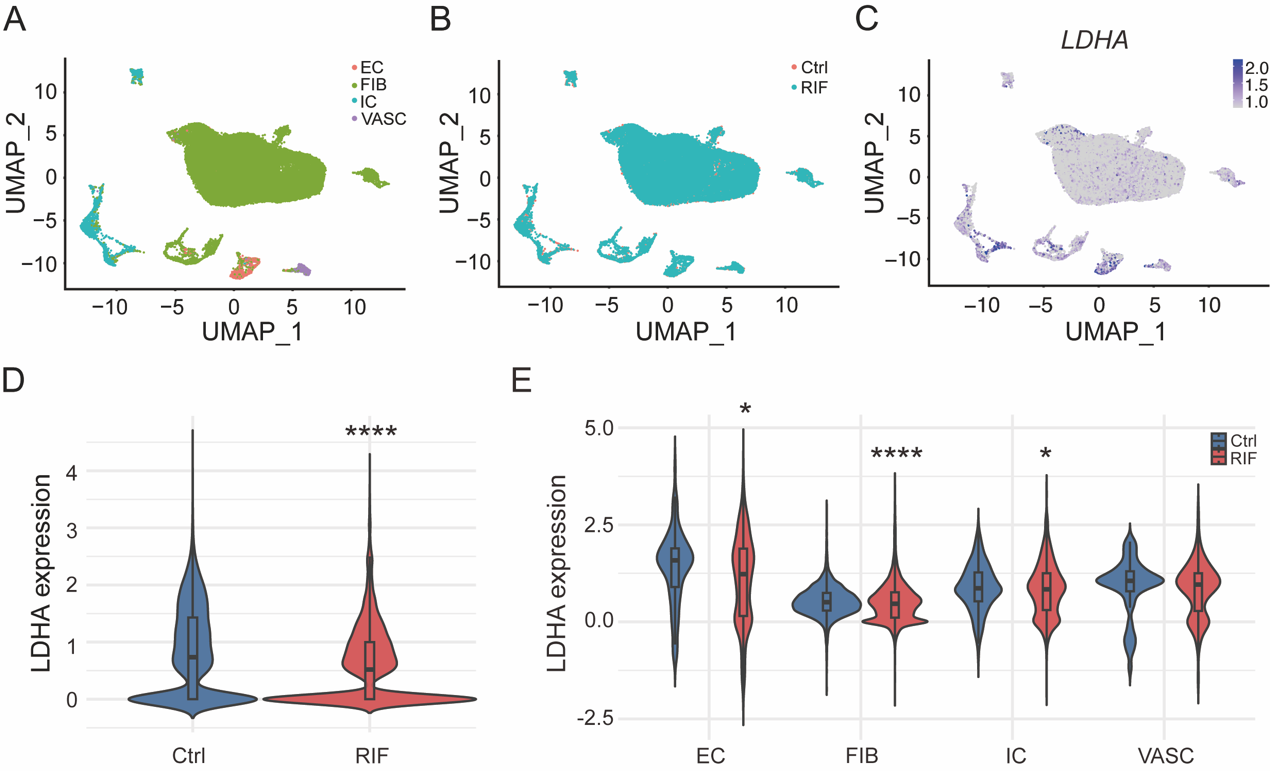
Figure S1. Single-cell RNA sequencing analysis of endometrium in healthy and RIF patients.**
(A) UMAP visualization of four major cell populations in endometrial tissue: epithelial cells (EC), fibroblasts (FIB), immune cells (IC), and vascular cells (VASC), colored by cell type.
(B) UMAP plot of cell distribution colored by condition: control (Ctrl) and recurrent implantation failure (RIF).

(C) Feature plot showing *LDHA* expression across the UMAP space in endometrial cells.

(D) Violin plot comparing *LDHA* expression in all endometrial cells between Ctrl and RIF groups. *****p* < 0.0001.

(E) Violin plot showing the difference in *LDHA* expression stratified by cell type from Ctrl and RIF groups. **p* < 0.05, *****p* < 0.0001.


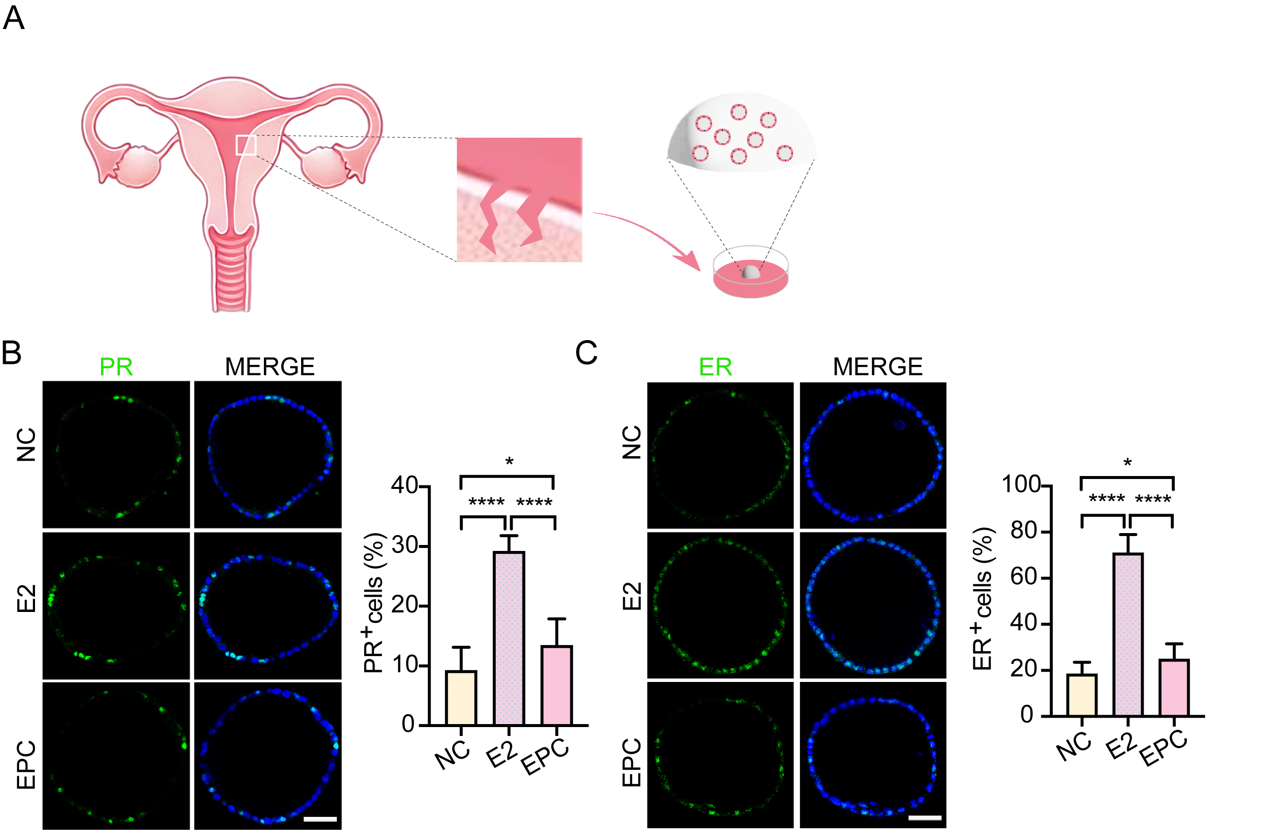


**Figure S2. Construction of EMOs that simulate menstrual cycle responses.**
(A) Schematic illustration of human EMOs generation from endometrial biopsies. Endometrial epithelium tissue fragments were isolated and subsequently cultured in a 3D Matrigel-based system.
(B-C) Representative immunofluorescence staining of PR (B) and ER (C) in EMOs treated with vehicle control (NC), estradiol (E2), or estradiol + progesterone + 8-Bromo-cAMP (EPC). Nuclei were counterstained with DAPI. Scale bar, 50 μm. Quantification of PR-positive and ER-positive cells is shown in the corresponding bar graphs (right panels). **P* < 0.05, *****P* < 0.0001.

**
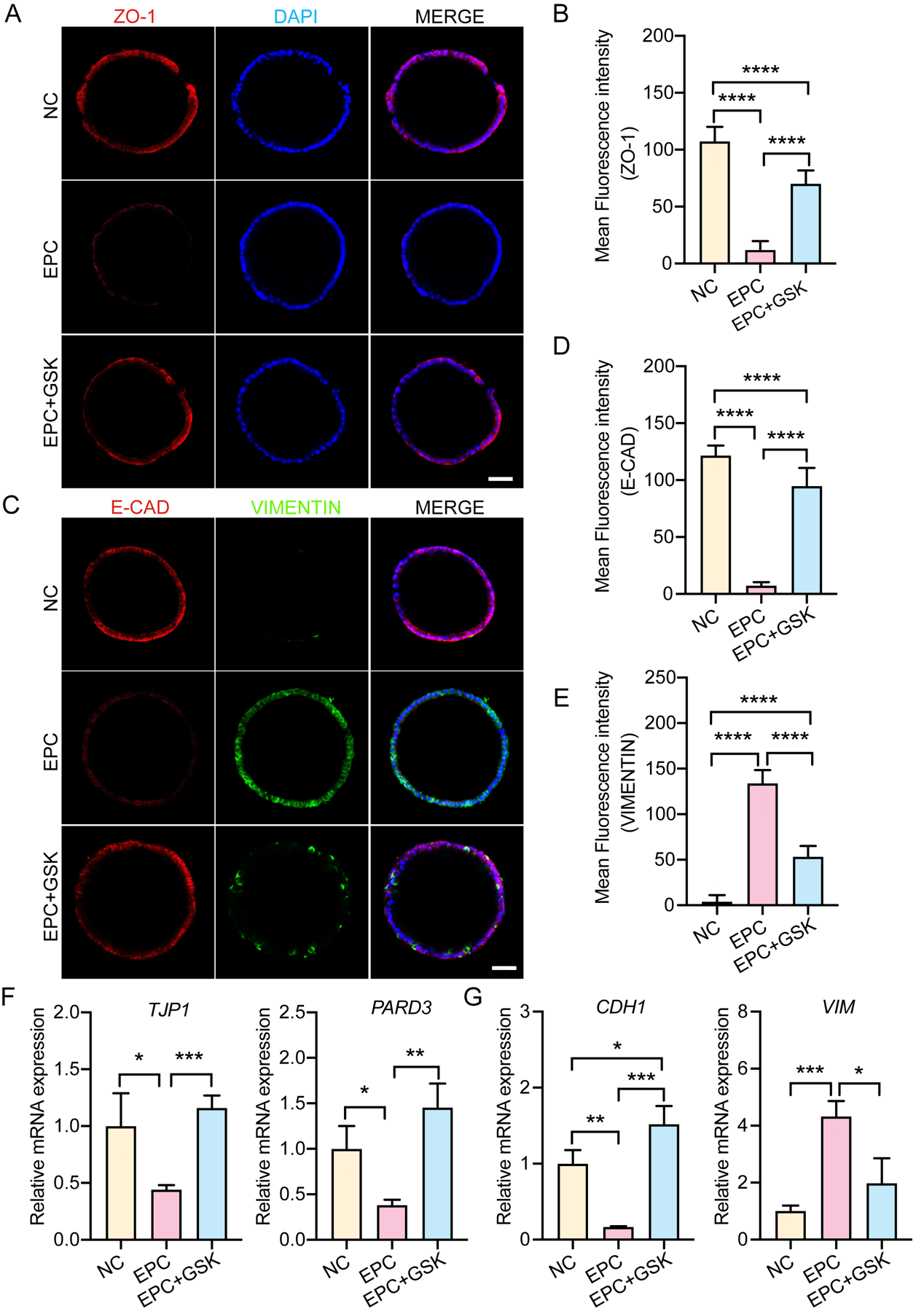
Figure S3. Pharmacological inhibition of LDHA prevents EPC-induced tight junction disruption in EMOs.**

(A-B) Representative immunofluorescence images and mean fluorescence intensities statistics of ZO-1 in EMOs under different treatments. EPC + GSK, EPC combined with LDHA inhibitor GSK 2837808A treatment. Scale bar, 50 μm. n = 12.

(C-E) Immunofluorescence staining and statistics of E-CAD and VIMENTIN under the different treatment conditions. Scale bar, 50 μm. n = 12.

(F-G) qRT-PCR analysis of polarity-related genes (*TJP1* and *PARD3*; panel F), and EMT markers (*CDH1*/E-cadherin and *VIM*/Vimentin; panel G).

**P* < 0.05,***P* < 0.01, ****P* < 0.001, *****P* <0.0001. **
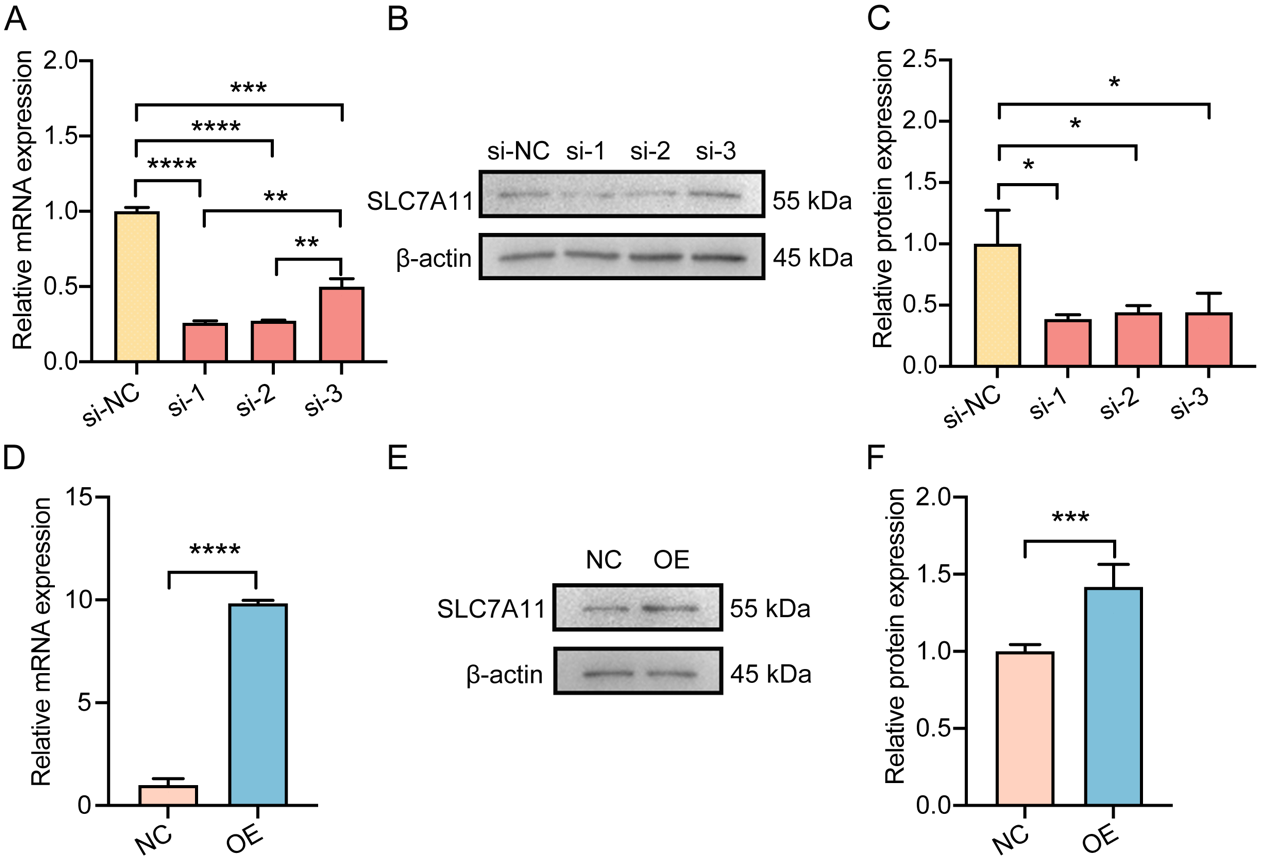
Figure S4. Validation of SLC7A11 knockdown and overexpression efficiency in Ishikawa cells.**
(A) Relative mRNA expression levels of *SLC7A11* determined by qRT-PCR after siRNA-1 (si-1), siRNA-2 (si-2) and siRNA-3 (si-3) transfection.
(B-C) WB analysis of SLC7A11 protein expression levels after siRNA transfection. n = 3.
(D) Relative mRNA expression of *SLC7A11* assessed by qRT-PCR after overexpression plasmids (OE) transfection.
(E-F) WB showing increased SLC7A11 protein levels in overexpression plasmids transfection. n = 3.
**P* < 0.05, ***P* < 0.01, ****P* < 0.001, *****P* < 0.0001.


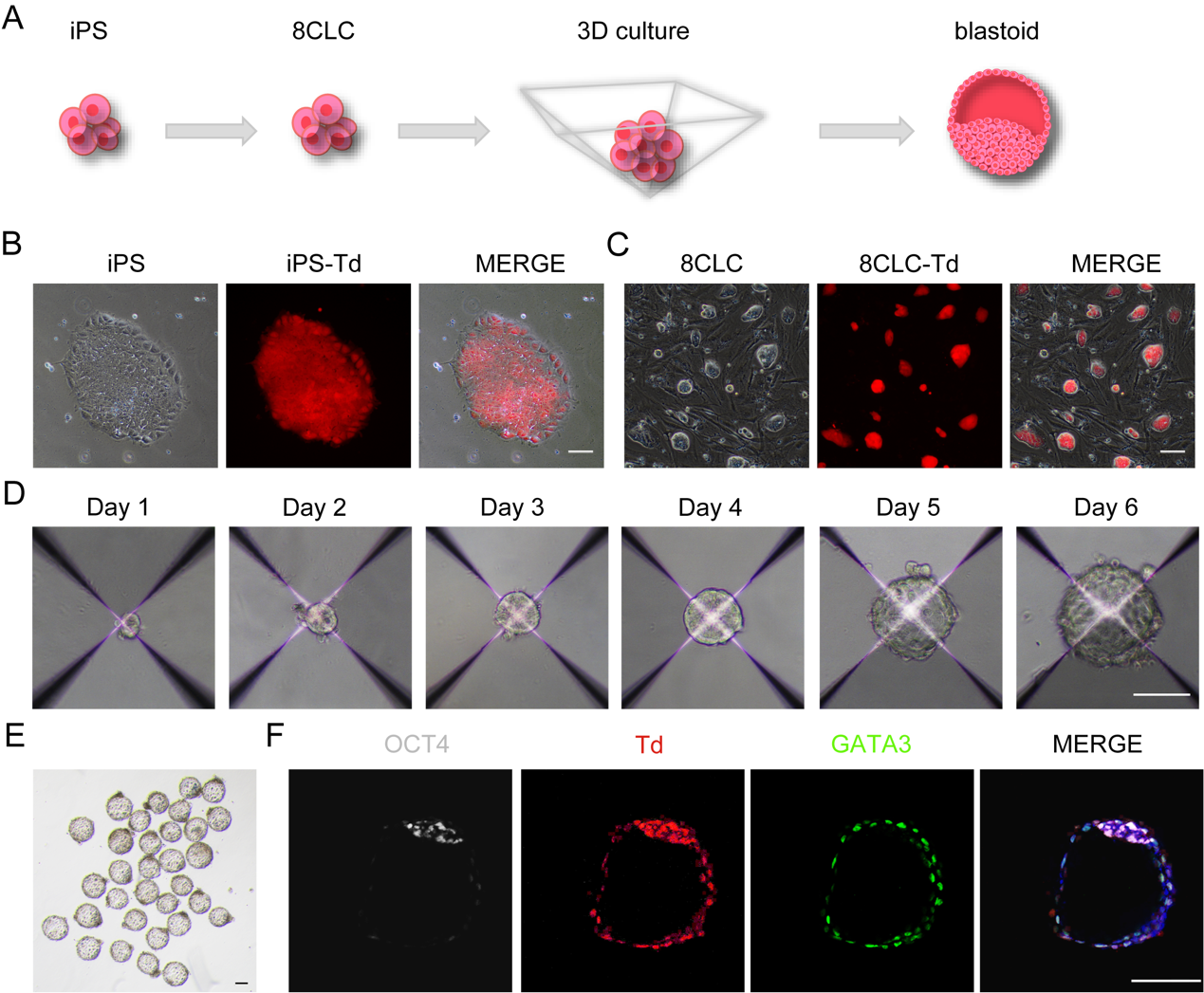


**Figure S5. Generation of human blastoids from iPS-derived 8CLCs.**(A) Scheme of generating blastoids from 8CLCs. iPSCs were first induced into 8CLCs, followed by aggregation in a 3D culture system to form blastoid structures.
(B-C) Fluorescence labeling of iPSCs (B) and 8CLCs (C) using tdTomato (Td). Representative bright-field and fluorescent images show efficient labeling of cells. Scale bars, 100 μm.
(D) Representative brightfield images of the stepwise morphological changes during blastoid formation in AggreWells from Day 1 to Day 6. Scale bars, 100 μm.
(E) Representative image of a population of formed blastoids after 6 days in culture. Scale bars, 100 μm.
(F) Immunofluorescence staining of blastoid sections for OCT4 (pluripotency marker), Td (tracking label), and GATA3 (trophectoderm marker). Merged image shows spatial separation of lineages, indicative of blastocyst-like organization. Scale bars, 100 μm.

Table 1. Baseline characteristics of the study population.

| **Feature** | **Control (n=6)** | **RIF (n=6)** | **Statistics (t/X2)** | **P value** |
| --- | --- | --- | --- | --- |
| Age (years) | 29.0±3.06 | 29.83±4.88 | t=-0.37 | 0.72 |
| BMI (kg/m2) | 21.0±1.59 | 21.17±3.47 | t=-0.11 | 0.91 |
| Number of embryo transfers | 1 | ≥4＋ | - | - |

Table 2. Primer sequences of siRNA in this study.

| **Name** | **Sequence (5′- 3′)** |
| --- | --- |
| siRNA-1 | F: GGAAGAGAUUCAAGUAUUAtt  R: UAAUACUUGAAUCUCUUCCtt |
| siRNA-2 | F: GGAAGUCUUUGGUCCAUUAtt  R: UAAUGGACCAAAGACUUCCtt |
| siRNA-3 | F: GAAUCUUCAUCUCUCCUAAtt  R: UUAGGAGAGAUGAAGAUUCtt |

Table 3. Details of antibodies used in this study.

| **Products** | **Sources** | **applications** | |
| --- | --- | --- | --- |
| Rabbit Anti-GATA3 | Abcam | IF | |
| Mouse Anti-Oct-4 | Santa Cruz | IF | |
| Rabbit Anti-Vimentin | ABclonal | IF | |
| Mouse Anti-E-cadherin | ABclonal | IF | |
| Mouse Anti-ZO-1 | Proteintech | IF | |
| Rabbit Anti-L-Lactyl Lysine | PTM BIO | IF, WB | |
| Rabbit Anti-H3K14la | PTM BIO | WB | |
| Rabbit Anti-H3K18la | PTM BIO | IF, WB | |
| Rabbit Anti-β-ACTIN | Bioworld | WB | |
| Rabbit Anti-H3 | PTM BIO | WB | |
| Rabbit Anti-SLC7A11 | ABclonal | WB | |
| HRP-conjugated Affinipure Goat Anti- Rabbit | proteintech | WB | |
| HRP-conjugated Affinipure Goat Anti-Mouse | proteintech | WB | |
| Donkey anti-Mouse, Alexa Fluor 555 | Thermo Fisher Scientific | IF | |
| Donkey anti-Rabbit, Alexa Fluor 488 | Thermo Fisher Scientific | IF | |
| Donkey anti-Mouse, Alexa Fluor 488 | Thermo Fisher Scientific | IF |  |
| Donkey anti-Rabbit, Alexa Fluor 555 | Thermo Fisher Scientific | IF |  |
| Donkey anti-Mouse, Alexa Fluor 647 | Thermo Fisher Scientific | IF |  |

Table 4. Details of primer sequences of genes used for qPCR.

| **Primers** | **Sequences** (5'-3') |
| --- | --- |
| *hLIF-F* | GCCACCCATGTCACAACAAC |
| *hLIF-R* | CCCCCTGGGCTGTGTAATAG |
| *hAREG-F* | GTGGTGCTGTCGCTCTTGATA |
| *hAREG-R* | CCCCAGAAAATGGTTCACGCT |
| *hCDH1-F* | CGAGAGCTACACGTTCACGG |
| *hCDH1-R* | GGGTGTCGAGGGAAAAATAGG |
| *hVIM-F* | GACGCCATCAACACCGAGTT |
| *hVIM-R* | CTTTGTCGTTGGTTAGCTGGT |
| *hPARD3-F* | GGGGACGGCCACATGAAAG |
| *hPARD3-R* | TTCCAAGCGATGCACCTGTAT |
| *hTJP1-F* | CAACATACAGTGACGCTTCACA |
| *hTJP1-R* | CACTATTGACGTTTCCCCACTC |
| *hSLC7A11-F* | TGTGTGGGGTCCTGTCACTA |
| *hSLC7A11-R* | CAGTAGCTGCAGGGCGTATT |
| *hEPHA4-F* | TACACCCCACAGCAGAATGG |
| *hEPHA4-R* | AATGGATGATGGTGCTGCTTG |
| *hBCL6-F* | CGTGAGCAGTTTAGAGCCCA |
| *hBCL6-R* | CCGCAAATTGAGCCGAGATG |
| *hLDHA-F* | ATGGCAACTCTAAAGGATCAGC |
| *hLDHA-R* | CCAACCCCAACAACTGTAATCT |
| *hLDHB-F* | ACCAGGCCCTACTTGTCCTT |
| *hLDHB-R* | TTCATCAGCCAGAGACTTTCCC |
| *hβ-ACTIN-F*  *hβ-ACTIN-R* | CATGTACGTTGCTATCCAGGC |
|  | CTCCTTAATGTCACGCACGAT |
| *hGAPDH-F*  *hGAPDH-R* | TCGACAGTCAGCCGCATCTTCTTT  ACCAAATCCGTTGACTCCGACCTT |
